# The pelvic flexure separates distinct microbial communities in the equine hindgut

**DOI:** 10.1038/s41598-021-83783-z

**Published:** 2021-02-22

**Authors:** Kailee J. Reed, Isabelle G. Z. Kunz, Jessica A. Scare, Martin K. Nielsen, Philip J. Turk, Robert J. Coleman, Stephen J. Coleman

**Affiliations:** 1 Animal Sciences, Colorado State University, Fort Collins, CO 80521 USA; 2 Cell and Molecular Biology Program, Colorado State University, Fort Collins, CO 80521 USA; 3 M.H. Gluck Equine Research Center, Department of Veterinary Science, University of Kentucky, Lexington, KY 40546 USA; 4 Atrium Health, Charlotte, NC 28203 USA; 5 Animal and Food Sciences, University of Kentucky, Lexington, KY 40546 USA

**Keywords:** Genetics, Microbiology, Physiology, Anatomy, Animal physiology

## Abstract

As hindgut fermenters, horses are especially dependent on the microbiota residing in their cecum and large intestines. Interactions between these microbial populations and the horse are critical for maintaining gut homeostasis, which supports proper digestion. The current project was motivated to determine if any features of the fecal microbiota are informative of the microbial communities from the cecum, ventral colon, or dorsal colon. Digesta from the cecum, ventral colon, dorsal colon and feces were collected from 6 yearling miniature horses. Microbial DNA was isolated and the microbiota from each sample was characterized by profiling the V4 region of the 16S rRNA. Principal coordinate analysis of the beta diversity results revealed significant (p = 0.0001; F = 5.2393) similarities between the microbial populations from cecal and ventral colon and the dorsal colon and fecal samples, however, there was little overlap between the proximal and distal ends of the hindgut. These distinct population structures observed in our results coincide with the pelvic flexure, which itself separates intestinal compartments with distinct roles in digestive physiology. An indicator species analysis confirmed the population differences, supported by the identification of several microbial families characteristic of the compartments upstream of the pelvic flexure that were not represented following it. Our data suggest that the fecal microbiota is not informative of the proximal hindgut but can provide insight into communities of the distal compartments. Further, our results suggest that the pelvic flexure might be an important anatomical landmark relative to the microbial communities in the equine large intestine.

## Introduction

The equine gastrointestinal tract (GI) is a complex organ system that can be divided into two main sections. The foregut, which consists of the stomach (glandular and non-glandular regions) and small intestine (duodenum, jejunum and ileum), is the primary site of enzymatic digestion and absorption of nutrients^1,2^. The hindgut, which consists of the cecum, large colon (ventral and dorsal), and small colon, is the primary site of fiber digestion and water reabsorption^3,4^. The hindgut accounts for approximately 62% of entire GI volume^3–5^. Horses are monogastric herbivores with diets consisting of high fiber and insoluble carbohydrates. Horses do not possess the enzymes required to digest the dietary fiber they consume and instead rely on the production of volatile fatty acids by fermentation in their hindgut to generate energy^6^. This fermentation and associated energy production depend directly on the activity of resident microbes^7^.

The cecum and large colon support the majority of the microbial populations that exist in the equine GI^6,8^. Collectively, these populations of microorganisms are referred to as an animal’s microbiota, a term often used interchangeably with “microbiome” which refers to the collection of genes and genomes contributed by the community of microorganisms^9^. Horses, in particular, rely heavily on the microbial communities in their GI tract. The symbiotic nature of the interaction between host and microbiota in the equine GI is critical for proper physiology and performance of the horse^10^. Proper GI function relies on maintaining dynamic equilibrium between the host and the microbial community constituents. Disruption of the microbial communities within the GI has been associated with metabolic syndrome^11^, colitis^7,12^, and several other GI dysbioses in horses and other mammals. Since most of the GI compartments are not directly or easily accessible, fecal samples may provide an effective proxy to monitor the microbiota in those compartments.

Sampling of feces is commonly used for investigations of the gut microbiota because the sampling is non-invasive, and it is relatively convenient to acquire fresh material for analysis. The Human Microbiome Project suggested that fecal samples are sufficiently representative of the microbial communities present across the various intestinal compartments in the humans to serve as an effective proxy^13^. Additional published studies have supported the utility of fecal microbial analysis as a proxy of the gut communities^14^ and for discovery and monitoring of biomarkers related to gut health^15,16^. Fecal samples informing about gut health and disease has been thoroughly reviewed previously^17^ as well as the importance of the collection methods for microbiome studies^18,19^. Methods for study of the equine microbiome using fecal samples have also been reviewed^20^ and many studies are published which use fecal samples to examine the link between GI microbiota and horse health^8,21–24^. Several investigations have been conducted comparing the microbial populations of equine fecal samples with different segments of the equine GI. Studies which relied on PCR-based methods both identified differences between fecal samples and different hindgut compartments, suggesting caution when making inferences based on fecal analysis^25,26^. Other studies which relied on sequencing of the 16S rRNA gene demonstrated that the small and large intestine have distinct microbial profiles compared to one another and that there are considerable differences between the compartments of the hindgut^27,28^. Another sequence-based GI survey demonstrated that the microbial communities of the hindgut become more distinct from that observed in the feces as they proceeded proximally along the GI tract towards the cecum^29^. Most recently, a study of the GI tract microbiota in Mongolian horses supported the conclusions of distinct differences between the small and large intestines and that direct sampling of intestinal compartments was more accurate than indirect analysis using fecal samples^30^. Previous investigations have not specifically examined the variation in microbial profiles in the separate segments of the hindgut or if community features are shared amongst other areas.

Here, we characterized the microbial populations of three main compartments in the equine hindgut and compared those to microbiota of the feces. Using robust statistical analyses not previously applied for studies of the equine GI microbiota, the current study provides new insight of the equine hindgut microbial populations and identifies features that are indicative of the hindgut GI compartments.

## Results

A total of 2,163,951 sequencing reads were generated from the 24 samples sequenced. Following quality control and feature table construction, 4461 total features (mean of 485 frequencies per feature) were identified. For downstream analysis, the feature table was sampled at a depth of 59,157 features per sample, from which 65.61% (1,419,768) of the original sequences and all 24 samples were retained for analysis.

### Diversity metrics

As the digesta from the proximal to distal compartments of the hindgut, Shannon Diversity Index values seemed to increase slightly (averaged Shannon Diversity Index’s based upon body site: cecum: 8.01; right ventral colon: 8.04; right dorsal colon: 8.10; feces: 8.41). However, there was no significant differences between the GI compartments or feces.

**Figure 1:**
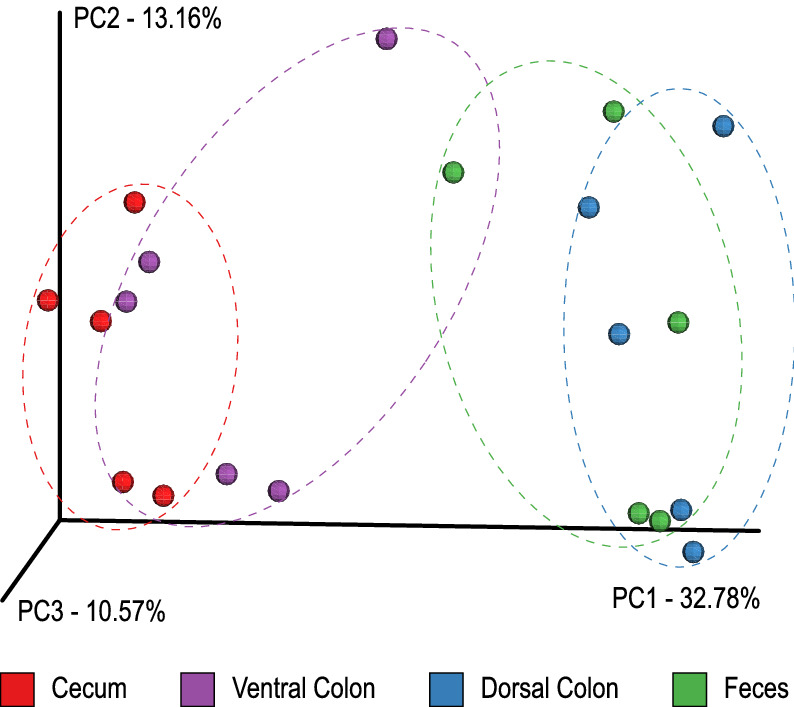
Principal coordinate analysis of gut microbial populations from cecum (red), right ventral colon (green), right dorsal colon (orange) and feces (blue).

Beta diversity was assessed using Bray–Curtis dissimilarity values. A principal coordinates analysis was done using the Bray–Curtis dissimilarity matrix in order to visualize the community’s differences (Fig. 1), showing clustering of communities based upon body site. The cecum and ventral colon microbial communities were clustered together, while the dorsal colon and fecal communities clustered together. The F-test was highly significant, confirming that the microbial community is different among body sites (p = 0.0001). Pairwise comparisons were also tested and all these comparisons with cecum were significant (p = 0.03125), indicating these populations differ, while dorsal to fecal, dorsal to ventral and fecal to ventral comparisons were not significant (p = 0.09375; p = 0.09375; p = 0.0625, respectively), indicating more similar microbial communities. Dispersion testing of the PERMANOVA results revealed that there is insufficient evidence to suggest the four body sites differed with respect to dispersion (p = 0.4648).

### Taxonomic classifications

**Figure 2:**
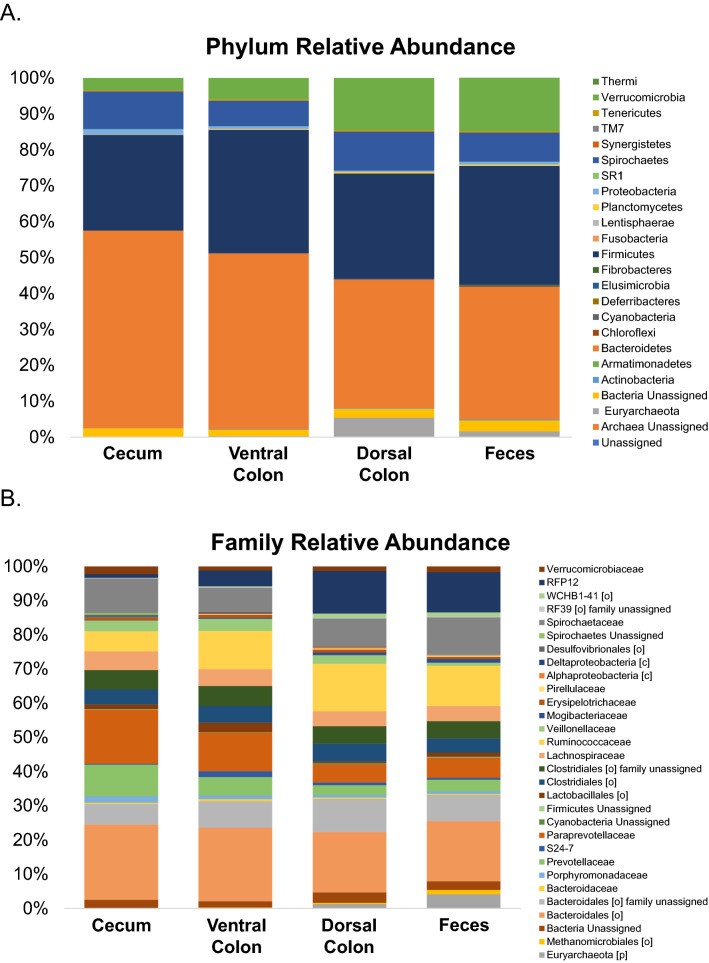
Relative abundance of microbial communities identified in the cecum, right ventral colon, right dorsal colon and feces, assigned to the phylum (**A**) and family level (**B**). For the family relative abundances, this figure depicts the top 30 features in the category, The complete list of families identified is available in Table S2.

The total number of features identified in each GI compartment increased from the cecum to the dorsal colon, with the largest number of features identified in the fecal samples. There was also a corresponding increase in the number of features which were shared by a specific GI compartment and the feces. The microbial phyla identified were principally Bacteroidetes and Firmicutes, with representation of Spirochaetes, Verrucomicrobia and Euryarchaeota as well. There was an apparent shift of microbial populations before and after the pelvic flexure with a large decrease (~ 50% to ~ 30%) in Bacteroidetes identified in the dorsal colon and fecal samples compared with samples from the cecum and ventral colon. The phylum Euryarchaeota increased in abundance in the samples following the pelvic flexure. The taxonomic differences before and after the pelvic flexure were in concordance with the beta diversity clustering results. The phyla and family relative abundances presented in Fig. 2A,B respectively. The corresponding taxonomic data for the individual horses is available in Tables S1 and S2.

### Indicator species analysis

To further understand the microbial community structures of the equine hindgut, we applied an indicator species analysis to identify representative features from each GI compartment. We used absolute counts at the family level due to the small number of reads mapped to the species (~ 27% of sequence reads were identified at the species level) and genus (~ 38% of sequence reads were identified at the genus level) levels. By contrast, ~ 60% were identified at the family level. For this reason, species was referred to as family in our analyses.

The first step of the analysis was identification of features at the ‘family’ level which are characteristic of a ‘single’ body site. The analysis revealed five families (Prevotellaceae, Paraprevotellaceae, unclassified Planctomycetes, unclassified Spirochaetes, unclassified Synergistales) to be indicators of the cecal community and two families (S24-7 and unclassified Lactobacillales) to be indicators of the right ventral colon community. No families were significant indicators of the right dorsal colon or fecal communities. This could be due to the number of animals that were available for this study. Table 1 displays the specific families, indicator values and associated p-values.

**Table 1:** Families identified to be most significant indicators based on single body sites, which include the indicator value (IV) and p-value for each. (*o* order, *p* phylum).

Body site	Family	IV	p-value
Cecum	Prevotellaceae	0.6792	0.0196
	Paraprevotellaceae	0.6567	0.0435
	Planctomycetes (p)	0.8606	0.0278
	Spirochaetes (p)	0.9071	0.0195
	Synergistales (o)	0.8511	0.0195
Ventral colon	S24-7	0.7379	0.0195
	Lactobacillales (o)	0.7017	0.0435

The second step of the analysis was to identify families that were indicative of microbial communities shared between body sites. The combined comparisons were also intended to identify signatures shared between the feces and the individual hindgut compartments. The indicator value formula was modified and the randomization test re-run. There was one family (unclassified Synergistales) identified as an indicator of the cecum and right ventral colon communities, three families (unclassified WCHB1-41, unclassified Euryarchaeota, unclassified Methanomicrobiales) were indicative of the right dorsal colon and feces and two families (unclassified Anaerolineae, Pirellulaceae) identified for the right ventral colon, right dorsal colon, and feces. The indicator values and associated p-values of these results are displayed in Table 2.

**Table 2:** Families identified to be most significant indicators based on combinations of body sites, which include the indicator value (IV) and p-value for each. (*o* order, *p* phylum, *c* class).

Body sites	Family	IV	p-value
Cecum & ventral colon	Synergistales (o)	0.9285	0.0396
Dorsal colon & feces	WCHB1-41 (o)	0.9155	0.0385
	Euryarchaeota (p)	0.9451	0.0385
	Methanomicrobiales (o)	0.8995	0.0424
Ventral/dorsal colons & feces	Anaerolineae (c)	0.9520	0.0424
	Pirellulaceae	0.9616	0.0385

## Discussion

The present study investigated the composition of microbial communities residing in distinct compartments of the equine hindgut (includes the cecum, large colon, and small colon) and compared them with fecal samples obtained from the same animal in order to determine similarities and differences between those communities. The data comparing the fecal microbiota to the microbiota from different segments of the equine GI tract has not yet conclusively determined it’s utility for that purpose^27,29,31^. These studies report differing results in regard to which compartments had similar microbial populations when compared to the fecal samples. Thus, our goal of this study was to further determine what features, if any, of the fecal microbiota were informative for understanding the microbiota from the cecum, ventral colon, and dorsal colon. Our results reveal some shared aspects of the microbial communities at the four sites tested but also support the conclusion that each compartment comprises a unique ecosystem which supports a distinct microbiota. We demonstrate the presence of shared community features across each GI location and the fecal sample. A majority of the taxonomic features identified by the sequencing data were shared by all four test sites (cecum, ventral colon, dorsal colon, and feces). The closer the compartment is to the terminal end of the digestive tract, the more shared taxonomic features there are between the feces and the GI compartments.

There are several results from our analysis which reinforce the conclusion that the various compartments of the equine hindgut support a microbial population which is specific to that region. The observed differences seem to correlate with the shifting physiological roles of each compartment in the process of digestion. The general metrics of these differences include changes in diversity and taxonomic abundances between the sampling sites. Alpha diversity increased, though non-significantly, from the cecum to feces indicating within sample diversity differences between the different intestinal sites corresponding to an increase in species richness (calculated Shannon Diversity Index value) along the length of the hindgut. The increase in alpha diversity corresponds with an increase in the total number of taxonomic features identified in each compartment as sampling proceeded from the proximal to distal end of the hindgut. Other published studies comparing the different compartments of the equine gut have reported data which reflects a similar trend in alpha diversity^28,32^. There are differences noted between the alpha diversity in our study and these others which may be due to host (breed and age), environmental (diet and management), or technical (sample collection and processing) factors. Additional study is required in horses to understand how these variables might impact analyses of the microbiome. Beta diversity, as visualized by PCoA generated using Bray–Curtis values, revealed clustering of the microbial communities from the different sampling sites. The first axis identified that the greatest contribution to separation between the samples was in fact body site. Further, there was a distinct separation of the communities identified in the cecum and ventral colon relative to those of the dorsal colon and feces (Fig. 1). The differences between body sites were statistically significant (p < 0.0001) as were the differences between cecum and the other samples with respect to community composition. The shift in beta diversity relative to body site is supported by observed differences in the taxonomic profiles derived from the different samples. The resulting taxonomic plots (Fig. 2A,B) reveal a unique distribution of the taxonomic classifications. Despite the differences between body site, it is also apparent that the cecal and ventral colon profiles are similar to each other, as are the dorsal colon and fecal profiles. As with the beta diversity results, there is an observable separation between the cecum and ventral colon relative to the dorsal colon and fecal samples. Anatomically, this distinction corresponds with the pelvic flexure.

The differences in community structure observed in our results agree with previously published comparisons of the microbiota from different compartments of the equine hindgut^27–29,33^. With some variation, corresponding to variation in sample processing, sequencing primer selection, and analytical approach, these studies all demonstrate a difference in community composition between the proximal and distal ends of the hindgut. As with our reported differences, the pelvic flexure sits between the compositional shifts reported by these other studies. Dougal et al. (2012) first described the differences between the cecal and right dorsal colon microbial communities and recognized that the populations shift corresponded to the pelvic flexure^33^. Similarly, Costa et al. (2015) reported changes in relative taxonomic abundance following the pelvic flexure and a study by Ericsson et al. (2016) revealed large dissimilarity between the cecum/ventral colon and the dorsal colon^27,28^. In both of these studies, the level of dissimilarity between the proximal and distal compartments appears less substantial than the dissimilarity observed between microbial communities of the small and large intestines, however it remains detectable. In our data, changes in the microbial populations of the various hindgut compartments seemed to be related to the pelvic flexure. Interestingly, the microbial changes observed relative to the pelvic flexure are detectable in each of the described studies even with sampling from different population of animals. The sampled populations used are vastly different in terms of geographical location, diet, breed, age, gender, and management practices. Separately, these factors have been demonstrated to have significant impacts on composition of the gastrointestinal or fecal microbiota in other species, including horses^34,35^. The fact that the data from these different studies all display a similar pattern of community structure across the various segments of the hindgut may indicate the importance of digestive physiology in determining composition of the GI tract microbiota. One possible explanation of the consistent pattern of differences between hindgut compartments could be a so called “core” microbiota or a set of requirements constituents for a functional microbial community as suggested by Dougal et al.^33^. Another possible explanation for the similar results observed across studies, despite the differences in animal subjects, sampling methods, and protocols used, could be the host’s control over whether particular members of the microbiota are retained or removed from different compartments of the gastrointestinal tract. Several published studies have provided evidence that the host “shapes” the microbiota present in its GI tract through a variety of physiological mechanisms. These include secretion of immunological mediators^36^, post-transcriptional regulation of gene expression^37,38^, cell differentiation and inflammation^39^ and overall gut homeostasis.

Taxonomic classifications and measures of alpha and beta diversity are capable of describing broad trends in microbial communities in terms of composition and differences which may be associated with important physiological or environmental parameters. In our data, these metrics indicate both the similarities and differences between the various compartments of the equine hindgut. These metrics do not, however, address the original premise of this study. Published studies have demonstrated that the fecal sample provides a reasonable representation of the distal hindgut compartments, but not the proximal ones^29^. These findings are contrasted with another study which seem to indicate that the fecal microbial population is similar to the population in the cecum^31^. Regardless of the conflicting conclusions, none of these previous studies have completely addressed the utility of a fecal sample for understanding the GI tract microbiota. In our study, we applied an indicator species analysis approach^40,41^ to identify features of the microbiota which could help define the compartments of the hindgut and determine if any of these features were shared with the fecal sample. Indicator species analyses were developed in the context of ecological community investigations^40^ and have been adapted previously to the study of gut microbiota^42–44^. Our study is the first time that an indicator species analysis has been used with the equine gastrointestinal microbiota.

We first considered the intestinal compartments and fecal sample individually for our comparisons in order to identify family features from each community which were characteristic of sampling site—so called indicator families. There were more significant indicator families identified in the cecum compared to the other body sites followed by the right ventral colon. An increased number of indicator families present at the cecum and ventral colon may be associated with their central roles in microbial fermentation of the fiber and insoluble carbohydrates in the equine diet. Five indicator families were identified in the cecum and 2 identified in the right ventral colon. The Prevotellaceae family was identified as an indicator family in the cecum and has been associated with many functions but is primarily recognized for its role in the breakdown of the proteins and complex carbohydrates present in feedstuffs. This functional role is especially important in the rumen of cattle and sheep, and the hindgut of horses^45^. The indicator status of Prevotellaceae in the cecum aligns with its anatomical function as the primary site of fermentation and breakdown of complex carbohydrates which pass through undigested from the small intestine. Research of the equine cecal microbiota using culture-based methods also identified the Prevotellaceae family to be highly abundant^32,33,46^, which seems to support its status as an indicator of the cecal ecosystem. The confirmation of Prevotellaceae’s importance in the cecum by these methods also lends credibility to the more novel families identified in each compartment by our analysis. Another indicator family identified in the cecum, also found in previous studies^32^, was Paraprevotellaceae. Unfortunately, little is known about the functional consequences of this group in mammals. It is likely an interesting candidate for further investigation to better understand its potential impact on digestive physiology in the cecum. An unclassified family member from the order Lactobacillales was identified as an indicator family in the right ventral colon. Members of this order have been implicated in the final stages of carbohydrate fermentation with individual families branching into many distinct species and strains^47^. Like the cecum, the right ventral colon is an important site of fermentation in the equine hindgut, and the presence of Lactobacillales families aligns with physiological role. This order has also been demonstrated to naturally associate with mucosal surfaces (like those of the GI tract) and is known for the metabolism and fermentation of lactic acid^48^. The family S24-7 was also identified as an indicator family in the right ventral colon and has been demonstrated to inhabit the gut of other homeothermic mammals^49^. Individual members of this family can be differentiated by IgA-labeling^50,51^ which suggests a potential host-microbe interaction through the immune system. There were no indicator families identified in either the right dorsal colon or fecal samples. Following the individual comparisons, sites were combined in order to compare communities from the proximal and distal regions of the hindgut. This second round of the analysis identified three features as indicators of the distal hindgut. One of these, an uncharacterized family member of the WCHB1-41 order, is a eubacterium that has been identified previously in biofilms^52^. The presence of WCHB1-41 is difficult to detect and track, but further research in terms of how this order interacts with intestinal epithelium and microbes could be beneficial for the poorly understood communication pathway between host and microbe. Also identified as indicators of the distal hindgut region were two archaea groups (these are two of the three groups mentioned above—the third was WCHB1-41), an uncharacterized family of the Euryarchaeota phylum and an uncharacterized family of the order Methanomicrobiales. Euryarchaeota, a dominant phylum discovered in several other studies, has been associated with host and gut interactions^53–55^, but with very specific genus and species in other mammals. Members of the Archaea kingdom has been associated with function in the gastrointestinal tract of many different mammals including horses, cattle and sheep^56–58^.

The motivation of the current study was to determine if a fecal sample could provide insight into the microbial communities of the various compartments of the equine hindgut. More importantly, we hoped to establish which features of the fecal microbiota were informative to understanding of microbial communities within the GI. As determined by taxonomic analysis, beta diversity and the indicator species analysis, fecal samples would seem to provide a good metric for investigations of the distal hindgut. Specifically, uncharacterized families from the order WCHB1-41, the order Methanomicrobiales, and the phylum Euryarchaeota may provide the most direct insights. Additional work will be required to determine if these similarities are consequential or informative when using a fecal sample to monitor the compartments of the distal hindgut. Specifically, are changes to the microbiota in the dorsal colon and other compartments reflected in changes to the fecal microbiota. The limit of a fecal sample for the communities of the hindgut seems to extend up to the pelvic flexure. Proximal to that point, the fecal communities display distinct differences from the communities in the ventral colon and cecum. This supports the conclusion that fecal samples are not informative of the proximal hindgut. Curiously, our data suggest a role of the pelvic flexure and potentially a physiological aspect from the host as an important component of the curation of microbial communities in different parts of the GI, which should be investigated further.

## Methods

### Animal subjects and sample collection

Digesta and fecal samples were collected postmortem from 6 yearling Miniature breed horses housed and managed at the University of Kentucky in Lexington, Kentucky. Animal care and handling methods were conducted in accordance with the guidelines and regulations established by the Animal Welfare Act administered by the USDA Animal and Plant Health Inspection Service. The study design and analysis parameters conform to the ARRIVE recommendations for animal research. All animal protocols were reviewed and approved by the University of Kentucky Institutional Animal Care and Use Committee (protocol number 2012-1046). Animals were maintained with a body condition score between 5.5 and 6.5 on a mixed grass hay pasture with ad libitum access to food and water with no deworming treatment prior to sample collection. The miniature horses were sampled from Population-S^59^ (a well-known herd within the equine research community that is used for studying equine health as it relates to parasite infections) and had an equal distribution of males (n = 3) and females (n = 3). The miniature horses were euthanized following the approved protocol for reasons unrelated to gastrointestinal disease between March and July 2016. Gut digesta and fecal samples were collected within one-hour postmortem. Sample collections proceeded as follows. The GI tract was removed and each intestinal compartment, the cecum, right dorsal colon, and right ventral colon, were identified and segregated. Digesta were emptied from each compartment, mixed and a sub-sample was collected for our analysis. Fecal material was collected from the rectum. Gut contents and fecal matter were placed in sterile 50 mL conical tubes and stored at − 20 °C.

### DNA isolation

Collected samples were thawed on ice and homogenized in sterile cups. Two hundred and fifty milligrams (0.25 g) of the homogenized sample was used for the extraction protocol. Microbial DNA was isolated using the PowerSoil DNA Isolation Kit (Qiagen, Venlo, Netherlands) according to the manufacturer’s recommended protocol with two minor alterations. The two modifications were (1) the addition of a 65 °C incubation for 10 min prior to vortexing the samples after addition of the lysis solution, and (2) increasing the centrifuge speed to 13,000 *g* for each centrifugation step. DNA quality (purity, quantity, integrity) was evaluated by spectrophotometry, using the NanoDrop 1000 (ThermoFisher Scientific Inc., Waltham, MA, USA), and PCR amplification of the V4 region of the 16S rRNA gene using the 515F/806R Earth Microbiome Project (EMP) standard primer set. Thermocycler settings and reaction mixtures can also be found under the protocols and standards section of the EMP website (http://www.earthmicrobiome.org/protocols-and-standards/dna-extraction-protocol). Samples were deemed acceptable if the DNA concentration was above 25 ng/μl, the 260/280 ratio was 1.8 or higher, and amplicons following PCR were approximately 300–350 base pairs.

### 16S rRNA library preparation and next generation sequencing

Sequencing libraries were generated by targeting the V4 region of the 16S rRNA gene according to the EMP protocol (http://www.earthmicrobiome.org/protocols-and-standards/16s/). Each sample was amplified in triplicate, pooled and visualized by gel electrophoresis to verify the correct insert size of 300–350 base pairs. Amplicon sequencing was performed on an Illumina MiSeq platform using V3 chemistry and generated 2 × 151 bp sequence reads (SeqMatic LLC, Fremont, CA, USA).

### Data processing and statistical analysis

Demultiplexing of the pooled samples was completed using the MiSeq Reporter Software System (Illumina, San Diego, CA, USA) using a golay error-correcting barcode associated with the forward primer sequence. Raw sequence files were imported into Qiime2 version 2017.11. The DADA2 pipeline was used to concatenate the forward and reverse pairs of each read, to correct for phiX controls, and to detect and remove chimeric sequences^60^. The resulting feature table was used to generate a phylogenetic tree for diversity metric analysis using the FastTree2 method^61^ and for taxonomic assignments using GreenGenes (v13.8) reference database by training a BLAST + classifier^62^.

Alpha diversity was evaluated by calculation of the Shannon Diversity Index, a quantitative measure of both the amount and abundance of different species within a sample^63^. Significance of intestinal site variable effect on alpha diversity was tested using a pairwise Kruskal–Wallis method comparing each of the body sites. Beta diversity was analyzed using PERMANOVA. This multivariate technique uses permutations of distances between horse and body site combinations computed with Bray–Curtis dissimilarity to assess the relative similarity of community structure between samples^64^. We first applied a square root transformation to the abundance (count) data due to the extreme range of values. Then, the Bray–Curtis dissimilarity was computed for each pair of horse-body site combinations. Due to the fact that equivalent dispersion among groups is an assumption of PERMANOVA, we also tested the multivariate homogeneity of group dispersions (variances)^64^.

### Indicator species analysis

Indicator species represent community features which characterize a group of samples and has commonly been used for ecological comparisons^40^. Indicator species analysis combines information about the abundance of a feature (species, genus, family, etc.) and the faithfulness of occurrence of a feature in a particular group^41^. Our ‘groups’ were the different body sites we sampled from (cecum, ventral colon, ventral colon, feces). An indicator value was calculated for each family in each group based off faithfulness (Eq. 1 below = ${F}_{ir}$: measure of constancy of presence of a family in a particular body site among the 6 horses) and exclusiveness (Eq. 2 below = ${E}_{ir}$: measure of the family’s specificity to a particular body site to the exclusion of other body sites). These can be computed with the equations below, where y_ijr_ is the abundance for the ith body site (i = 1,2,3,4), the jth horse (j = 1,2,3,4,5,6) and the r^th^ family (r = 1, 2, …, 87), ${\stackrel{-}{y}}_{ir}$ is defined as the mean abundance over horses for family (r) in body site (i), and I_ijr_ is an indicator function denoting the presence or absence of family (r) in body site (i) for horse (j).

1 $${F}_{ir}=\frac{{\sum }_{j}{I}_{ijr}}{6}$$

2 $${E}_{ir}=\frac{{\stackrel{-}{y}}_{ir}}{{\sum }_{i}{\stackrel{-}{y}}_{ir}}$$

The indicator value (IV_ir_) for each family (r) in body site (i) is defined by the Eq. 3 below. The highest IV_ir_ for a family among the body sites was taken to be the overall indicator value for the family and is denoted IV. If the indicator value for each family in the body site approaches 1, this means this family is a good indicator for a particular body site. However, if the indicator value approaches 0, this means the family is a poor indicator for that body site.

3 $${IV}_{ir}=\sqrt{{E}_{ir} x {F}_{ir}}$$

Analysis was performed in R (v 3.5.0) using packages indicspecies^65^ and vegan^66^. The data matrix imported consisted of 87 families across 24 body sites $\times$ horse combinations, with body site being a factor with four levels and six horses. Using a randomization test (Monte Carlo), body sites were randomly shuffled within horse (block) 9999 times and IV_ir_ was computed each time in order to obtain a p-value for our observed value. To control the Type I error rate, we ran the Benjamini–Hochberg method at a false discovery rate of 0.05 to find the families with significant indicator values.

## Supplementary Information

Supplementary Information

## Data Availability

All raw high-throughput sequencing data described here has been submitted to the NCBI Sequence Read Archive and is available under BioProject number PRJNA631014.
